# Value of using ultrasonic shears in reducing seroma formation after axillary lymph node dissection in breast cancer patients

**DOI:** 10.1186/s43046-024-00248-w

**Published:** 2024-12-18

**Authors:** Yousra Mohsen Elshoura, Ahmed Refaat, Basma Hussein Abdelaziz Hassan, Philobater Bahgat Adly Awad, Mohamed Wael Ahmed, Sherif Mokhtar, Emad Salah El din Khalaf

**Affiliations:** 1 General Surgery Department, Faculty of Medicine, Cairo University, Cairo, Egypt; 2 General Surgery Department, Faculty of Medicine, Ain Shams University, Cairo, Egypt

**Keywords:** Axillary lymph node dissection, Drainage time, Seroma formation, Breast cancer

## Abstract

**Background:**

Axillary lymph node dissection (ALND) is an essential step in the management of breast cancer. ALND is conventionally performed using radio frequency electrosurgery. The post-operative complications of utilizing such energy (such as prolonged drainage time, seroma, or infection) lead to prolonged recovery. Hence, it may delay the initiation of adjuvant chemo/radiotherapy for this critical category of patients. Using ultrasound shears provides a wide spectrum of tissue effects via mechanical oscillation. The absence of an electric circuit in ultrasound shears reduces thermal injury and accordingly cellular damage.

**Objective:**

Comparing utilization of ultrasound shears in axillary lymph node dissection to conventional radio frequency electrosurgery in terms of operative time, post-operative drainage amount and days, post-operative pain, the incidence of seroma or infection, and lymph node yield.

**Methods:**

This study is a randomized control trial. It includes 56 breast cancer ALND cases performed in conjunction with either BCS or MRM; being upfront surgery cases or post-neoadjuvant therapy cases, 28 patients underwent ALND using ultrasound shears and 28 underwent ALND using radio frequency electrosurgery.

**Results:**

The mean age of the study population was 51 ± 11.7 years, with a mean BMI of 39. The mean operative time in the ultrasound shear group was 29.4. ± 7.6 min and 31.6 ± 5.1 min in the conventional group. The mean amount of drainage in the ultrasound shear group was 319.6 ± 75.4 ml and 407.5 ± 75.2 ml in the conventional group. The mean drainage days in the ultrasound shear group were 8 ± 1 day and 12 ± 2.2 days in the conventional group. Seroma formation was recorded in 6 of the ultrasound shear groups and 9 in the conventional group. Seroma followed by infection was found in 10% of the ultrasound shear group versus 21% in the conventional group. Seroma formation and wound infection were significantly related to the conventional group (*p*-value = 0.01).

**Conclusion:**

Our study recommends the utilization of ultrasound shears in ALND as it is a safe and accurate method that allows faster post-operative recovery with shorter drainage time and lower incidence of seroma or infection, without affecting operative time or lymph node yield.

**Trial registration:**

Trial no.: PACTR202402831197428. Date of approval: 19/02/2024

## Introduction

Axillary lymph node dissection (ALND) remains a substantial step in the management of breast cancer with infiltrated axillary lymph nodes [10]. Such cases require to be followed by adjuvant therapy in order to gain systemic control of the invasion. Complications of ALND such as seroma, lymphedema, or infection should not be under-estimated as they can delay the initiation of adjuvant therapy when a maximum of 8 to 12 weeks is required in between both steps. It is of crucial importance to pass this interval promptly so as not to lose grounds to recurrence [29].

Of all ALND complications, seroma constitutes a stumbling block through the gap interval which may lead to other complications and may itself lengthen the interval [27]. Up till today, the pathophysiology of seroma remains a matter of debate. A multifactorial hypothesis has been accepted to regard seroma as an exudative collection of acute inflammatory reaction to surgical and thermal trauma at the cellular level accompanied with deficient sealing of vessels and lymphatic ducts [8].

With a wide range of incidence from 3 to 85%, seroma risk factors have been studied thoroughly in literature. Age, breast size, and BMI are considered to be the main factors affecting seroma formation [14].

Several studies have proposed different strategies in the attempt to reduce seroma formation. They include obliteration of dead space [21], spraying tetracycline [11], applying fibrin sealants [30], using somatostatin receptor such as octreotide [2], and delaying shoulder mobilization [26]. In a meta-analysis by Kuroi et al. [14], most of the mentioned attempts were evaluated and were not found to be of significant contribution in reducing their complication.

Closed suction drains have served since 1947 as loyal raconteurs to post-operative collections of dead spaces beneath the sutures [19]. Currently, low vacuum suction drains are the most common surgical method used to drain seroma instead of their repeated aspirations. However, they require meticulous surveillance and maintenance to keep patency, encompass a degree of discomfort, and hinder physical activity [15]. Prolonged drain maintenance has even been debated to cause more seroma and infection [28]. Recently, the trend has been to keep it as short as possible, and strategies differ between different centers [20].

Several meta-analyses have demonstrated the pivotal role of surgical technique and devices in the formation of seroma; the more radical the procedure is and the longer the operative time is, the more the seroma is and hence the drainage time and hospital stay Pan et al. [22], Clymer et al. [4], and Huang et al. [12].

Radio frequency electro surgery was first introduced as an efficient device for ALND, owing to its potential to achieve fast and easy hemostasis through the high emanated heat. Direct, high thermal energy was later disputed for its traumatic devitalization of tissues and inadequacy to seal lymphatics. This was revealed in a study by Porter et al. [23] that recorded a 38% incidence of seroma in electro surgery group in contrast to 13% for the scalpel dissection group, concluding that electro surgery can efficiently reduce intraoperative blood loss at the expense of increasing the incidence of seroma.

Having made a valuable stride at reducing intraoperative blood loss, another was ambitioned regarding reducing seroma. Ultrasonic harmonic shears can adequately provide both vascular and lymphatic sealing more by breaking hydrogen bonds which further reduces the inflammatory response [31]. Furthermore, the vibrating property makes hemostasis more efficient and the dissection more precise. This presents obvious superiority in ALND because there are more branch vessels and important nerves in the dissection plane [12].

## Patients and methods

This study was conducted between 1st of January 2021 and 1st of May 2021. The study protocol was reviewed and permitted by the institutional research and ethics committee.

This study includes 56 breast cancer patients; twenty-eight patients had axillary lymph node ultrasound-shear dissection, and twenty-eight had conventional radio frequency electric dissection.

### Inclusion criteria

Patients with breast cancer and pathological axillary lymph nodes are operated upon for ALND, either upfront or post neoadjuvant chemotherapy (NACT), as a part of breast conservation surgery (BCS) or modified radical mastectomy (MRM).

### Exclusion criteria

Metastatic breast cancer cases were excluded in the study.

### Pre-operative assessment

Patients presenting to the breast clinic with suspicious breast lumps were subjected to triple assessment in the form of clinical examination, sono-mammography, and tru-cut biopsy. When proven to have breast cancer, metastatic work-up and immuno-histochemistry on the biopsy (ER and PR receptors, HER2NEU, and Ki67) were completed. The patient’s data was presented to our breast clinic multidisciplinary team (composed of the following: Surgical Oncology, Medical Oncology, Radiotherapy, Radiology, and Pathology Consultants), and patients who were categorized clinically as node positive (N1-3) both by palpation and U/S examination were eligible for ALND.

### Randomization

Patients were divided into 2 groups via block randomization, where the first 28 cases presented were allocated as the “US shear” group, and the next 28 cases were allocated as the “Conventional” group.

### Operative

Standard level I and II axillary lymph node dissection was performed among both groups where:

Ultrasound shear group: axillary dissection and hemostasis were achieved using a harmonic focus device; the same device was used to seal vessels and lymphatics.

Conventional group: axillary dissection and hemostasis were achieved using monopolar diathermy device; vessels were ligated using vicryl 3-0.

Intercosto-brachial nerve was identified in all axillae and cut sectioned near the origin. Operative time was calculated from the opening of clavipectoral fascia till drain insertion.

Both groups had separate vacuum 18 F drains inserted in the axillary space. In case MRM was performed, quilting sutures between pectoralis minor and upper skin flap were taken to separate dissected breast compartment from axillary space.

In the MRM cases, quilting sutures were applied between the breast and axillary compartments to minimize dead space and reduce drainage, but in BCS cases, this was avoided to preserve the cosmetic outcome.

### Post-operative care

Patients were educated about the suction drains daily amount monitoring and evacuation, discharged (if no hemodynamic complications interfered) with drain in situ by the end of their second day, and instructed to send their daily drainage amounts through phone. Cases were called back to return to the department and remove their drains when the drain recorded less than 30 ml for 2 consecutive days.

Post-operative pain was assessed during the return–visit using the Numerical Rating Scale (NRS) shown in Table 1.

**Table 1:** Visual Numerical Rating Scale [7]

Rating	Pain level
0	No pain
1–3	Mild pain (nagging, annoying, interfering little with ADLs)
4–6	Moderate pain (interferes significantly with ADLs)
7–10	Severe pain (disabling, unable to perform ADLs)

*ADLs* Activities of daily living

A 1-month follow-up was achieved to evaluate if any complications (infection, seroma, or hematoma) have occurred and managed at that time frame.

Pathologies of the included study cases have all been revised for lymph node yield.

### Statistical analysis of data

Statistical analysis was conducted using SPSS 22nd edition; continuous variables were presented in mean ± SD and compared using the Mann-Whitney *U* test, while categorical variables were presented in frequency and percentage and compared using the chi2 test. Continuous variables were correlated using the Spearman correlation matrix. Any *p*-value < 0.05 was considered significant.

## Results

### General baseline characters

A total of 56 breast cancer patients were included in our final analysis. Group A (US Shear) was allocated 28 cases, and the same for group B (Conventional) baseline characters are described in Table 2. Mean age and BMI were similar in both groups. Group A had 10 cases with comorbidities, while group B had 4 cases. Tumor and lymph node stage distribution did not significantly differ between both groups nor did the percentage of cases that received neoadjuvant chemotherapy. Immuno-histochemistry (IH4) was not significantly different in both groups as well as the percentage of cases operated upon for BCS or MRM.

**Table 2:** Baseline character distribution

		A: US shear		B: Conventional		*P*-value
		Mean	SD	Mean	SD	
Age		51.0	11.0	51.0	12.7	0.99
BMI		38.7	8.6	39.0	8.4	0.69
		N	%	N	%	
Comorbid		10	35.7%	4	14.3%	0.06
T stage	T1	0	0.0%	0	0.0%	0.3
	T2	4	14.3%	0	0.0%	
	T3	14	50%	14	50%	
	T4	10	35.7%	14	50%	
N stage	N0	0	0.0%	2	7.1%	0.38
	N1	13	46.4%	12	42.9%	
	N2	14	50%	14	50%	
	N3	1	3.6%	0	0.0%	
IH4	LA	14	46.4%	10	32.1%	0.59
	LB	9	28.6%	10	32.1%	
	Her2	5	14.3%	7	25%	
	TN	0	0.0%	1	3.6%	
Neoadjuvant		12	42.9%	10	35.7%	0.58
Procedure	BCS	16	57.4%	18	64.3%	0.59
	MRM	11	39.4%	10	35.7%	
	Radical	1	3.2%	0	0.0%	

*BMI* Body mass index, *IH4* Immuno-histochemistry, *LA* Luminal type A, *LB* Luminal type B, *Her 2* Her 2 neo positive, *TN* Triple negative, *BCS* Breast conservation therapy, *MRM* Modified radical mastectomy

The baseline character distribution did not differ significantly between both groups as shown in Table 2.

### Primary outcomes

A significant difference was found regarding the total drain volume, where group A had a mean of 319.6 ml in contrast to 407.5 ml for group B. This volume difference did not appear on the first 48 h of drainage, but appeared on further days, summing up to a total drainage days mean of 8 days for group A versus 12.5 days for group B as described in Table 3. Higher rates of seroma were recorded in group B, with a total of 9 (31%) cases versus 6 (20%) for group A, where the higher rates were accounted for the higher incidence of infection in group B.

**Table 3:** Primary outcomes

0		A: US shear		B: Conventional		*P*-value
		Mean	SD	Mean	SD	
Total drain volume		319.6	75.4	407.5	75.2	< 0.0001
Drain volume 48 hr.		285	40.5	302	20	0.02
Drainage days		8.0	1.0	12.5	2.2	< 0.0001
		*N*	%	*N*	%	*P*-value
Seroma	Seroma only	3	10%	3	10%	0.01
	Seroma followed by infection	3	10%	6	21%	
	Total	6	20%	9	31%	

### Secondary outcomes

Post-operative pain records had a mean score of 5.7 (moderate) for group A and 5.9 (moderate) for group B with no statistical significance. Mean lymph node yield was not affected between groups A or B. A mean operative time of 29.4 min was recorded for group A versus 31.6 min for group B which was not statistically significant as shown in Table 4.

**Table 4:** Secondary outcomes

Secondary outcome	A: US shear		B: Conventional		*P*-value
	Mean	SD	Mean	SD	
Post-operative pain	5.7	0.8	5.9	1.1	0.45
LN yield	14.4	3.4	13.1	4.7	0.17
Operative time	29.4	1.0	31.6	5.1	0.16

*LN Yield* lymph node yield

### Confounding factors

During our data analysis, some baseline factors were found to affect the primary outcome of both groups. Age was significantly associated with an increased amount of drain post-operatively with *p*-value 0.001, also BMI significantly affected the total drain amount post-operatively with *p*-value < 0.0001 described in Table 5. Cases that received neoadjuvant chemotherapy recorded less amount of drainage volume in both groups (*p*-value 0.04) as shown in Table 6.

**Table 5:** Age and BMI affecting total drain amount

Variable	Spearman correlation coefficient	*P*-value
Age	0.43	0.001
BMI	0.62	< 0.0001

*BMI* Body mass index

**Table 6:** Neoadjuvant chemotherapy NACT, procedure type, and co-morbidity relation to total drain amount

		Drain amount (A)		Drain amount (B)		*P*-value
		Mean	SD	Mean	SD	
Neoadjuvant	Upfront	419.2	42.7	422.7	20.7	0.04
	Post NACT	357.5	35.8	378.2	24.6	
Procedure	BCS	430.0	98.7	435.8	82.7	0.079
	MRM	365.0	87.5	376.9	80.3	
Co-morbidity	No	357.8	106.4	390.7	94.4	0.051
	Yes	431	37	410.7	47.8	

*Post NACT* Post neoadjuvant chemotherapy, *BCS* Breast conservation surgery, *MRM* Modified radical mastectomy

## Discussion

The introduction of ultrasound dissectors in ALND by Galatius et al. [9] and Lumachi et al. [16] proved lower cellular damage, better vessel sealing, hemostasis, and reduced seroma formation, eventually improving post-operative recovery and healing. However, studies that followed afterward have differed in their results.

As opposed to predicted, the use of ultrasound shears in our study did not pose any lengthening in the operative time. The mean operative time for ALND using ultrasound shears was 29.4 ± 7.6 min versus 31.6 ± 5.1 for conventional ligation and electrocautery. Operative time was not significantly affected in several studies that included whole modified radical mastectomy (MRM) operations, where not just ALND but also flaps were executed using ultrasound shears. In a study by Khan et al. [13], a total of 76 MRM cases were performed by the same high-volume surgeon with experience in both devices, mean operative time for ultrasound shear cases was 191 ± 44 min versus 187 ± 36 min for electrocautery (*p*-value 0.499).

Exposure of surgeons to the energy devices prior to surgery has helped to avoid an increase in the operative time [12].

Ultrasound shears significantly reduced the total drain amount in our study, where the mean amount was 319.6 ± 75.4 ml for cases versus 407.5 ± 75.2 ml for controls (*p*-value < 0.0001). It has to be noted that there was no significant difference between drains of both groups in the first 48 h, consistent with the findings of Kuroi et al. [14]. This is supported by the findings in Damani et al. [6] and Rohaizak et al. [24], both studies reported less drainage amount for axilla dissected using ultrasound shears (mean 100 ml and 188 ml, respectively). The difference between our results and mentioned study results may be attributable to the variance of BMI between the populations studied; our sample BMI mean was 38.7 ± 8.6 versus 25 ± 5.2 for Rohaizak et al. [24].

Drain days for ultrasound shear cases, though recorded to have been reduced in our study and in literature, have been found to be of great variance between different studies. Our study recorded a mean of 8 days ± 1 for ultrasound shear dissected cases versus 12.5 days ± 2.2 for conventional dissection, which was close to the records of Memon et al. [17] (8.9 for ultrasound shears versus 13 for electrocautery), but far from Damani et al. [6] and Rohaizak et al. [24] recording only a mean of 2.8–3 days for ultrasound shears and 7 days for electrocautery. It should be mentioned that all studies in comparison adopted the policy of removing drains after 2 days of drainage of less than 30 ml according to Lin et al. [15]. Nonetheless, the variance in sample demographics between different study populations could be responsible for the difference in the findings recorded; eventually most studies have come to the same conclusion which is significant reduction in days of drain maintenance in cases dissected using ultrasound shears.

Post-operative pain scores did not differ between the 2 groups in this study, with most cases suffering moderate pain as most of the intercostobrachial nerves have been severed in the dissection of the 2 groups. There is no current clear consensus on dealing with the ICBN during axillary dissection, though a meta-analysis of RCTs by Sanjay et al. [25] demonstrates that preservation of the ICBN results in less sensory disturbance. Our study standardized all axillary dissection procedures into elimination of ICBN as to pose no risk of post-operative hypersensitivity, which would be of greater morbidity than axillary numbness. Though most studies recorded a significant enhancement of the pain experience post-operatively, Archana et al. [1] found no significant difference in pain scores for the first 4 post-operative days, only on the fifth day did they record a significance in favor of ultrasound shears (4 vs, 2,*p* = 0.002). However, it has to be noted that our mean score was 5.7 ± 0.8 which is still higher than most of other studies.

Ultrasound shears succeeded at reducing the incidence of seroma formation significantly from 31.7% of the conventional dissection cases to 20.6% in ultrasound shear cases (*p*-value 0.01). Unfortunately, 50% of the seroma cases in our studies passed into infection during their homestay. Similar findings were reported in Memon et al. [17] and Gambardella et al. [10], where Gambardella et al. [10] revealed an incidence of 16% using the newly introduced Thunderbeat® Olympus device, versus 24% for ultrasound shears. Thunderbeat newly introduced device utilizes both ultrasonic energy and bipolar radiofrequency enabling seal of vessels up to 7 mm.

However, the patient’s body mass index and undergoing neoadjuvant chemotherapy have to be taken into consideration as they affect the complexity of dissection and hence lengthen the operative time and eventually the drainage. Receiving neoadjuvant chemotherapy before operation has been stated as one of the confounding factors to decrease total drainage volume in our study. This could have been attributed to a fibrotic or xanthomatous response of tissue to chemotherapy that altered lymph node structure and drainage [5]. Also, the type of surgical procedure in our study was noticed to be another confounding factor to alter total drainage amount. Where all cases of modified radical mastectomy received quilting sutures to separate the breast from the axillary compartment and in return less axillary drainage volume, all cases that underwent breast conservation surgery received no less or a second-degree oncoplastic procedure without quilting sutures, if the axilla was accessed through the same incision. Unfortunately, conservation procedure subtypes were not included in data collection, in order to give an accurate judgement about the effectiveness of quilting. Quilting sutures was confirmed to decrease drainage amount and time in a systematic review and meta-analysis by Morarasu et al. [18].

All cases in this study were discharged on their 2nd post-operative day, where the reduction in drain days did not add up to decrease hospital costs. However, the use of ultrasound shears decreased the frequency of patient visits to outpatient clinics for dressing and decreased the frequency of repeated aspiration for seromas and the possible risk of secondary infection. It has to be taken in mind that ultrasound shears are 100 times more expensive than monopolar electrocautery diathermy, even though ultrasound shears can be sterilized and used more than once in low-income countries [3].

## Conclusion

Our study demonstrated that the usage of ultrasound shears managed to reduce the incidence of seroma formation (commonly anticipated after ALND surgery), total drainage amount, and days of drain maintenance without severing other parameters regarding operative time or post-operative pain.

## Data Availability

This study is a randomized control trial, conducted among breast cancer patients of Kasr Alainy Hospital of Cairo University. It includes 56 breast cancer ALND cases performed in conjunction with either BCS or MRM; being upfront surgery cases or post-neoadjuvant therapy cases, 28 patients underwent ALND using ultrasound shears and 28 underwent ALND using radio-frequency electrosurgery. The datasets used and/or analyzed during the current study are available from the corresponding author on reasonable request.
